# Biosynthesis, optimization, and multifunctional biomedical applications of gold nanoparticles mediated by *Streptomyces* sp. YJD18

**DOI:** 10.3389/fmicb.2025.1667928

**Published:** 2025-09-15

**Authors:** Zihan Lin, Niu Lijuan, Hailong Zhang, Haitao Shen, Wenzhong Hu, Wenyuan Yin, Fei Guo

**Affiliations:** ^1^Zhuhai College of Science and Technology, Guangdong, China; ^2^Microbiology Laboratory, College of Life Science and Technology, Xinjiang University, Xinjiang, China; ^3^Key Laboratory of Xinjiang Phytomedicine Resource and Utilization of Ministry of Education, College of Life Sciences, Shihezi University, Shihezi, China; ^4^Department of Anesthesiology, Baoshan Branch, Ren Ji Hospital, School of Medicine, Shanghai Jiao Tong University, Shanghai, China

**Keywords:** gold nanoparticles, *Streptomyces* sp. YJD18, cytotoxicity, wound healing, antioxidant activity, microbial synthesis

## Abstract

This study employed extracellular secretions from *Streptomyces* sp. YJD18, isolated from saline-alkali soil, to synthesize gold nanoparticles (AuNPs) using optimized conditions: 60 min boiling, 48 h mycelial suspension, pH 6.25, 1.0 mM chloroauric acid, 1:1 supernatant-to-water ratio, and 9.0 g mycelial wet weight. AuNP formation was confirmed by a yellow-to-ruby red color shift and a 525 nm UV–Vis absorption peak. TEM and SAED revealed irregular, polycrystalline spheres with good dispersity. Zeta potential (−11.6 mV) and DLS (20–30 nm) confirmed uniform surface properties and narrow size distribution. FTIR showed C–H and amide groups did not contribute to AuNP synthesis. The AuNPs exhibited dose-dependent cytotoxicity against cancer (cervical, lung, liver, breast) and normal (kidney, liver) cells, optimal wound healing at 30 μg/mL (58.07% at 48 h), and concentration-dependent DPPH scavenging (IC_50_: 12,419.00 μg/mL), suggesting biomedical potential.

## 1 Introduction

Gold nanoparticles (AuNPs) can be synthesized chemically, physically, or biologically (Huang et al., 2014; Kaliaraj et al., 2017). Traditional physical methods, such as laser ablation, require high energy, whereas chemical methods involve toxic reagents, limiting their environmental safety and broader application (Lee et al., 2020; Rónavári et al., 2021; Soto et al., 2021). These limitations have driven research into greener, biologically mediated synthesis approaches (Lengke et al., 2007; Shukla and Iravani, 2018).

Microbial synthesis, compared to physicochemical methods, offers mild reaction conditions, environmental friendliness, and superior biocompatibility. Microbial biomolecules act as natural reducing and capping agents, ensuring nanoparticle stability and preventing aggregation (He et al., 2007; Singh et al., 2016; Gengan et al., 2024). Among microbes, actinomycetes, particularly *Streptomyces* species, produce stable, diverse nanoparticles through intracellular and extracellular pathways, with extracellular methods offering better dispersity and simplified purification (Prakash et al., 2014; Boomi et al., 2020a; Kumari et al., 2020; Ielo et al., 2021). Such biologically synthesized nanoparticles have significant potential in biomedical applications (Bharadwaj et al., 2021; Lange et al., 2019).

Microorganisms adapted to extreme saline environments often exhibit unique metabolic pathways that are advantageous for the synthesis of bioactive compounds, including nanoparticles. Compared with commonly employed bacterial or fungal strains, actinomycetes are associated with lower risks of toxin and spore production, thus providing a safer platform for biosynthesis processes. In this study, we investigated the green synthesis of AuNPs using *Streptomyces* sp. YJD18, isolated from an extremely saline-alkaline environment. By optimizing synthesis conditions and evaluating anticancer, wound-healing, and antioxidant activities, we demonstrate the biomedical potential of this actinomycete-based approach as a novel strategy for producing functional metallic nanoparticles.

## 2 Materials and methods

### 2.1 Preparation of sterile mycelial supernatant from *Streptomyces* sp. YJD18

Soil samples were collected from the saline land around Wujiaqu City, Xinjiang Uygur Autonomous Region, and the collected soil samples were dried at 100°C, ground and sieved (400 mesh), the soil obtained after sieving was divided into portions of 5 g each, and spread in the bacterial Petri dish, after which, sterilized Actinobacillus solid medium was slowly poured into the Petri dish to cover the soil and put into the incubator for cultivation at a constant temperature of 28°C, and the cultivation temperature was 28°C. The incubation temperature was 28°C. Actinomycetes were isolated from the soil samples by utilizing the characteristic that actinomycetes are able to produce aerial mycelium while bacteria cannot, and were further cultured for identification.

YJD18 were cultured in Actinomyces liquid medium (Qingdao Hope Bio-Technology Co., Ltd) under constant shaking at 180 rpm and 28°C for 72 h. The actinomycete mycelia were harvested by centrifugation at 9500 rpm for 20 min and subsequently washed three times with sterile distilled water to ensure removal of residual media components. A 1 g wet biomass sample was suspended in 50 mL of sterile distilled water and incubated for 72 h at 180 rpm and 28°C following incubation, the cell-free supernatant was obtained by centrifugation at 10000 rpm for 20 min at 4°C. The resulting supernatant was filtered using JIAO JIE qualitative filter paper (Fushun City Civil Affairs Filter Paper Factory, China) to remove any remaining debris. This cell-free supernatant was subsequently used for AuNPs synthesis.

The identity of YJD18 was confirmed by 16S rRNA gene sequencing, and the sequence has been deposited in the NCBI GenBank database under the accession number PV291671.

### 2.2 Synthesis of AuNPs

A mixture of 50 mL sterile mycelial supernatant and 50 mL chloroauric acid solution (analytical grade, purchased from Benze Reagents) was prepared in a 250 mL Erlenmeyer flask, with the final concentration adjusted to 1 mM. The mixture was reacted in a boiling water bath for 30 min. Color changes (from light yellow to ruby red with noticeable transparency) were observed and recorded. Samples were taken every 10 min to measure absorbance at wavelengths between 200 and 800 nm using a UV–Vis spectrophotometer.

### 2.3 Optimization of AuNP synthesis conditions

To ensure the stability and high quality of the synthesized AuNPs, key synthesis parameters were systematically optimized using single-factor experiments. The formation and characteristics of AuNPs were monitored by UV–Visible (UV–Vis) spectroscopy. The parameters optimized included: Mycelial Biomass-to-Water Ratio: Ratios of 1 g:100 mL, 3 g:100 mL, 5 g:100 mL, 7 g:100 mL, and 9 g:100 mL were evaluated to determine the optimal biomass concentration for efficient nanoparticle synthesis; Chloroauric Acid Concentration: Final concentrations of 0.5 mM, 1.0 mM, 1.5 mM, and 2.0 mM were tested to identify the most effective precursor concentration; Supernatant-to-Water Volume Ratio: Ratios of 1:4, 2:3, 1:1, 3:2, 4:1, and 5:0 were examined to assess the impact of extracellular metabolites on nanoparticle formation; pH: A broad range of pH values (3.00, 5.00, 6.00, 6.25, 6.50, 7.00, 8.00, 9.00, and 10.00) was investigated to determine the optimal pH for AuNP biosynthesis; Resuspension Time: Mycelial resuspension durations of 24 h, 48 h, and 72 h were compared to optimize the extraction of active biomolecules involved in the reduction process; Boiling Time: Heating durations of 0, 10, 15, 20, 25, 30, 35, and 40 min were tested to evaluate the influence of thermal treatment on nanoparticle formation and stability.

### 2.4 Characterization of AuNPs

The synthesized AuNPs were characterized using the following analytical techniques: UV–Visible Spectroscopy (UV-3100PC, Shanghai Meipuda Instruments Co., Ltd.): Absorption spectra were recorded over the wavelength range of 200–800 nm with a resolution of 1 nm; Transmission Electron Microscopy (TEM) (FEI Talos F200X): The morphology of the particles was examined at an accelerating voltage of 200 kV under ambient conditions; Selected Area Electron Diffraction (SAED): The crystalline structure of the AuNPs was analyzed based on the diffraction pattern obtained during TEM imaging; Dynamic Light Scattering (DLS) and Zeta Potential Analysis (Malvern Zetasizer Nano ZS90): The particle size distribution, polydispersity index (PDI), and surface charge (zeta potential) of the AuNPs were measured; X-ray Diffraction (XRD) (Rigaku Ultima IV): The crystalline structure was determined using a scanning range of 20°–80°, with a step size of 0.02° and a scanning speed of 2°/min; Fourier-Transform Infrared Spectroscopy (FTIR) (Thermo Nicolet iS20): Functional groups associated with the AuNPs were identified in the spectral range of 400–4,000 cm^−1^, with a resolution of 4 cm^−1^; Samples were prepared by mixing 1 mg of AuNPs with 100 mg of KBr and pressing the mixture into pellets.

### 2.5 Cytotoxicity evaluation of AuNPs

#### 2.5.1 Cell culture

The cytotoxicity of the synthesized AuNPs was evaluated using a panel of human cell lines, including cervical cancer (HeLa), breast cancer (MDA-MB-231), lung cancer (A549), liver cancer (HepG2), and two normal cell lines: normal liver (WRL-68) and normal kidney (293 T). Key reagents used in this study included fetal bovine serum (FBS), penicillin–streptomycin antibiotics, trypsin, high-glucose Dulbecco’s Modified Eagle Medium (DMEM), RPMI-1640 medium (Biological Industries), MTT reagents (BIOFROXX), cisplatin (Bide Pharm), and dimethyl sulfoxide (DMSO; Beijing Coolaber Technology Co., Ltd.). HeLa cells were cultured in RPMI-1640 medium, whereas all other cell lines were maintained in DMEM supplemented with 10% FBS and 1% antibiotics. Cells were incubated at 37°C in a humidified atmosphere containing 5% CO₂ until reaching approximately 80% confluency.

#### 2.5.2 Cytotoxicity assay

The cytotoxicity of the synthesized AuNPs was evaluated using the MTT assay. The procedure was as follows: Cells at approximately 80% confluency were harvested using trypsinization and adjusted to a density of 1 × 10^5^ cells/mL; A volume of 100 μL of the cell suspension was seeded into each well of a 96-well plate and incubated for 24 h at 37°C in a 5% CO₂ atmosphere; Peripheral wells were filled with phosphate-buffered saline (PBS) to minimize evaporation; Various concentrations of AuNPs (0, 100, 200, 300, 400, 500, 600, 700, and 800 μg/mL) were added to the wells, followed by a 24-h incubation; The positive control was 30 μg/mL cisplatin, while the negative control consisted of sterile mycelial supernatant; After treatment, 100 μL of MTT solution (0.5 mg/mL) was added to each well and incubated in the dark for 4 h; The resulting formazan crystals were dissolved in 100 μL of dimethyl sulfoxide (DMSO) and gently shaken for 20 min; Absorbance was measured at 490 nm using a microplate reader.

Cell viability (%) was calculated using the following formula:


$$\text{Viability}(\%)=\frac{A_{s}}{A_{c}}\times 100$$

where *A_s_* is the absorbance of the treated group, and *Ac* is the absorbance of the control group.

Statistical analysis was performed using one-way ANOVA, and differences were considered statistically significant at *p* < 0.05.

### 2.6 Statistical analysis

In this study, the Statistical Package for the Social Sciences (SPSS) was used as the statistical tool to evaluate the significance of the research, specifically SPSS version 27.0. All quantitative data are presented as mean ± standard deviation (SD) from three independent biological replicates (*n* = 3). ANOVA was followed by *t*-test and *p* < 0.05 was considered statistically significant.

## 3 Results

### 3.1 Synthesis of AuNPs

**Figure 1:**
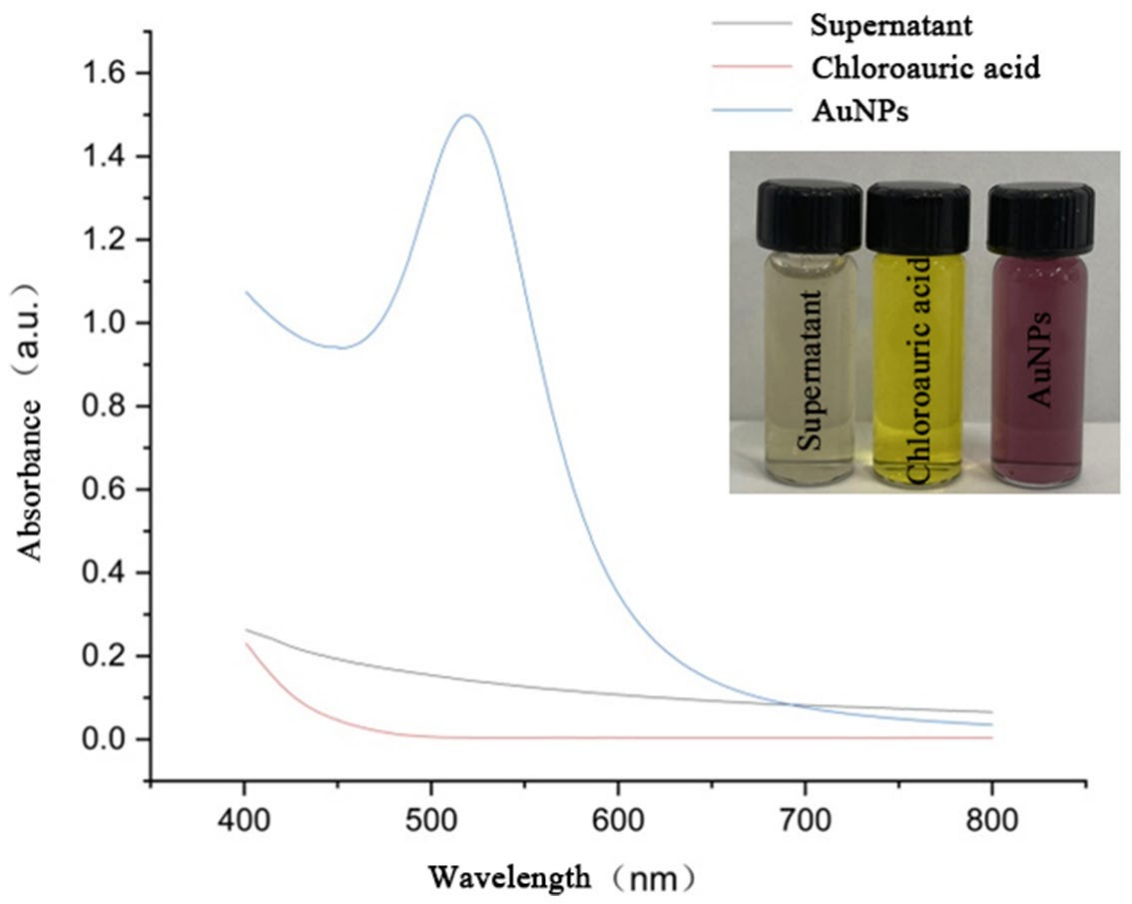
UV–Vis absorption spectra of sterile supernatant, chloroauric acid, and YJD18-synthesized AuNPs. The AuNPs exhibit a distinct surface plasmon resonance (SPR) peak at approximately 520 nm, confirming their successful formation. Inset: Photographic comparison of the three solutions, showing the color change from colorless (supernatant), to yellow (chloroauric acid), to ruby red (AuNPs).

As shown in Figure 1, during the reaction in a boiling water bath, the color of the solution gradually changed from light yellow to ruby red with noticeable transparency after approximately 10 min, indicating the successful synthesis of AuNPs (Inset of Figure 1). UV–Vis spectroscopy analysis showed a characteristic absorption peak at around 525 nm, further confirming AuNP formation. In contrast, no absorption peaks at 525 nm were observed in the control groups, suggesting the absence of AuNP synthesis.

### 3.2 Optimization of synthesis conditions

**Figure 2:**
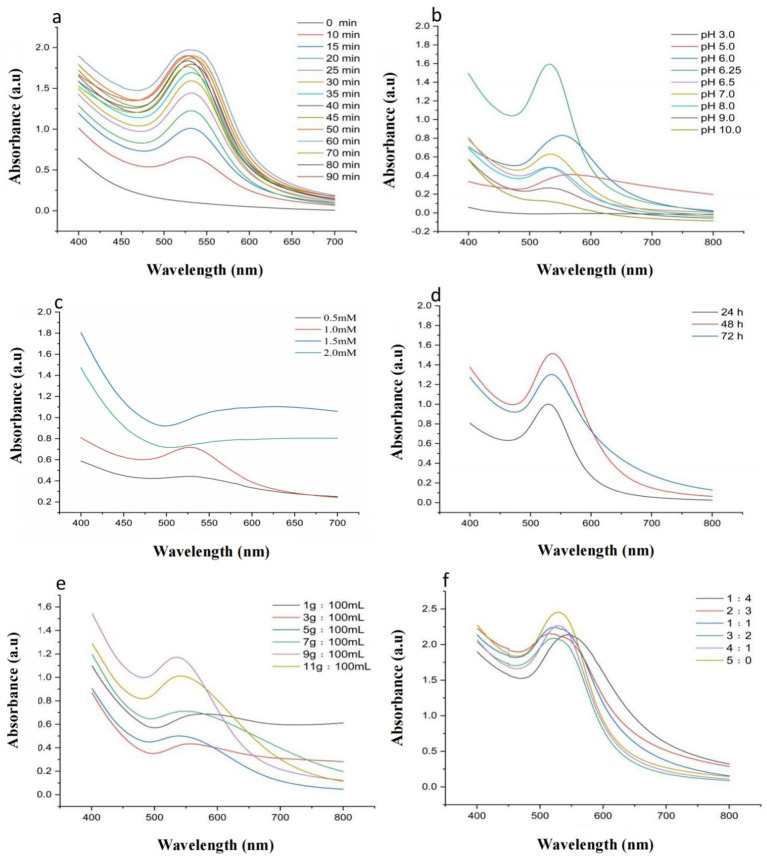
Optimization of Synthesis Conditions: **(a)** UV–Vis spectra of AuNPs synthesized using YJD18 supernatant under different boiling water bath durations. **(b)** UV–Vis spectra of AuNPs synthesized using YJD18 supernatant at various pH values. **(c)** UV–Vis spectra of AuNPs synthesized using YJD18 supernatant with different concentrations of chloroauric acid. **(d)** UV–Vis spectra of AuNPs synthesized using supernatant collected from YJD18 after different secondary fermentation durations. **(e)** UV–Vis spectra of AuNPs synthesized using supernatant derived from YJD18 cultures with varying mycelial biomass concentrations. **(f)** UV–Vis spectra of AuNPs synthesized at different volume ratios of YJD18 supernatant to distilled water.

As shown in Figure 2a, all groups except the 0 min group exhibited distinct absorption peaks in the experiment investigating the optimal boiling water bath duration. The maximum absorbance was observed at 60 min, and the peak position (~525 nm) corresponded to the characteristic surface plasmon resonance (SPR) absorption peak of AuNPs. Therefore, a boiling duration of 60 min was identified as optimal for AuNP synthesis.

Figure 2b illustrates the significant effect of pH on AuNP synthesis, with the highest absorption intensity observed at pH 6.25, indicating the greatest nanoparticle yield and optimal synthesis efficiency under this condition. The pH not only influences the reduction rate of gold ions but also directly affects the morphology and stability of the nanoparticles. Under mildly acidic conditions, the activity of bioreducing agents is more stable, promoting effective reduction of gold ions and uniform particle formation. In contrast, strongly acidic environments may damage biomolecular structures, inhibiting the reduction process and reducing synthesis efficiency. Although alkaline conditions can facilitate the reduction reaction, they often lead to particle aggregation, negatively impacting nanoparticle dispersity and uniformity (Ahmed et al., 2020; Hu et al., 2019). Therefore, controlling the pH of the reaction system plays a crucial role in achieving efficient and stable AuNP synthesis.

In Figure 2c, the influence of chloroauric acid concentration on AuNP synthesis is presented. A pronounced SPR absorption peak at approximately 525 nm was observed at a concentration of 1 mM. Further increasing the precursor concentration resulted in a gradual decrease of peak intensity, indicating that 1 mM was the optimal concentration for AuNP synthesis; Figure 2d shows the impact of mycelial biomass resuspension time on AuNP formation. The strongest absorption was recorded when cell-free supernatant, obtained after biomass resuspension for 48 h, was incubated with chloroauric acid. Thus, 48 h was considered the optimal resuspension duration. As depicted in Figure 2e, the biomass-to-water ratio also influenced AuNP synthesis. Among the tested ratios, the strongest SPR absorption peak appeared at 9 g:100 mL, indicating this condition was most favorable for nanoparticle formation; Finally, Figure 2f demonstrates the effect of the volume ratio between sterile supernatant and distilled water on AuNP synthesis. A 1:1 ratio resulted in an SPR peak position closely matching the typical AuNP absorption (~525 nm), whereas other ratios exhibited varying degrees of redshift. Hence, a ratio of 1:1 was identified as optimal for AuNP synthesis.

### 3.3 Characterization of AuNPs

#### 3.3.1 Transmission Electron microscopy (TEM) and X-ray diffraction (XRD)

**Figure 3:**
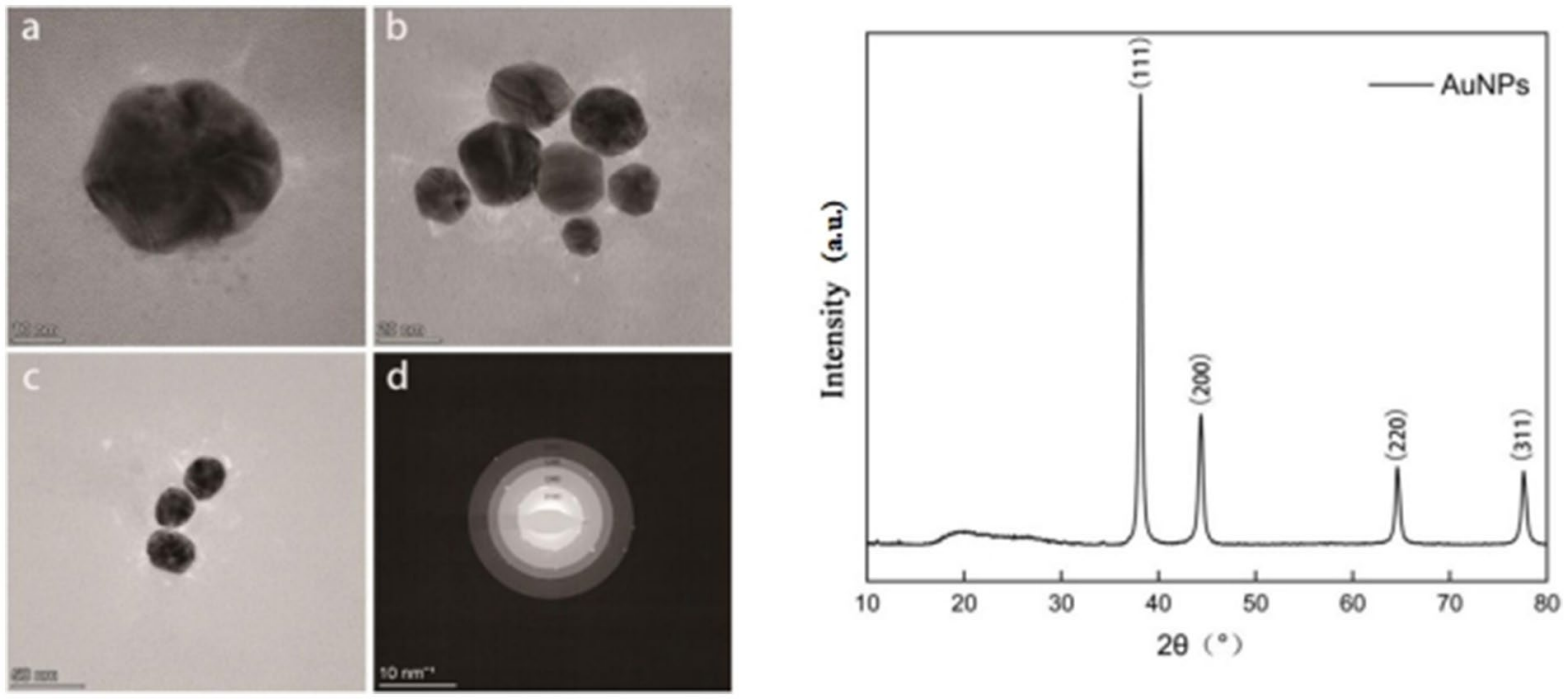
Transmission electron microscopy (TEM) images of synthesized AuNPs at different magnifications **(a–c)**; selected area electron diffraction (SAED) pattern **(d)**; and X-ray diffraction (XRD) pattern **(e)**.

As shown in Figures 3a–c, the synthesized AuNPs predominantly exhibited irregular spherical morphologies and displayed good dispersibility without significant aggregation. The selected area electron diffraction (SAED) pattern (Figure 3d) showed four distinct diffraction rings corresponding to the (111), (200), (220), and (311) crystallographic planes, confirming the polycrystalline nature of the synthesized AuNPs. The crystalline structure of the AuNPs was further confirmed by X-ray diffraction (XRD) analysis (Figure 3e), revealing characteristic diffraction peaks at 2θ values of 38.16°, 44.38°, 64.60°, and 77.60°, indexed, respectively, to the (111), (200), (220), and (311) planes of face-centered cubic (FCC) gold.

#### 3.3.2 Zeta potential and dynamic light scattering (DLS)

**Figure 4:**
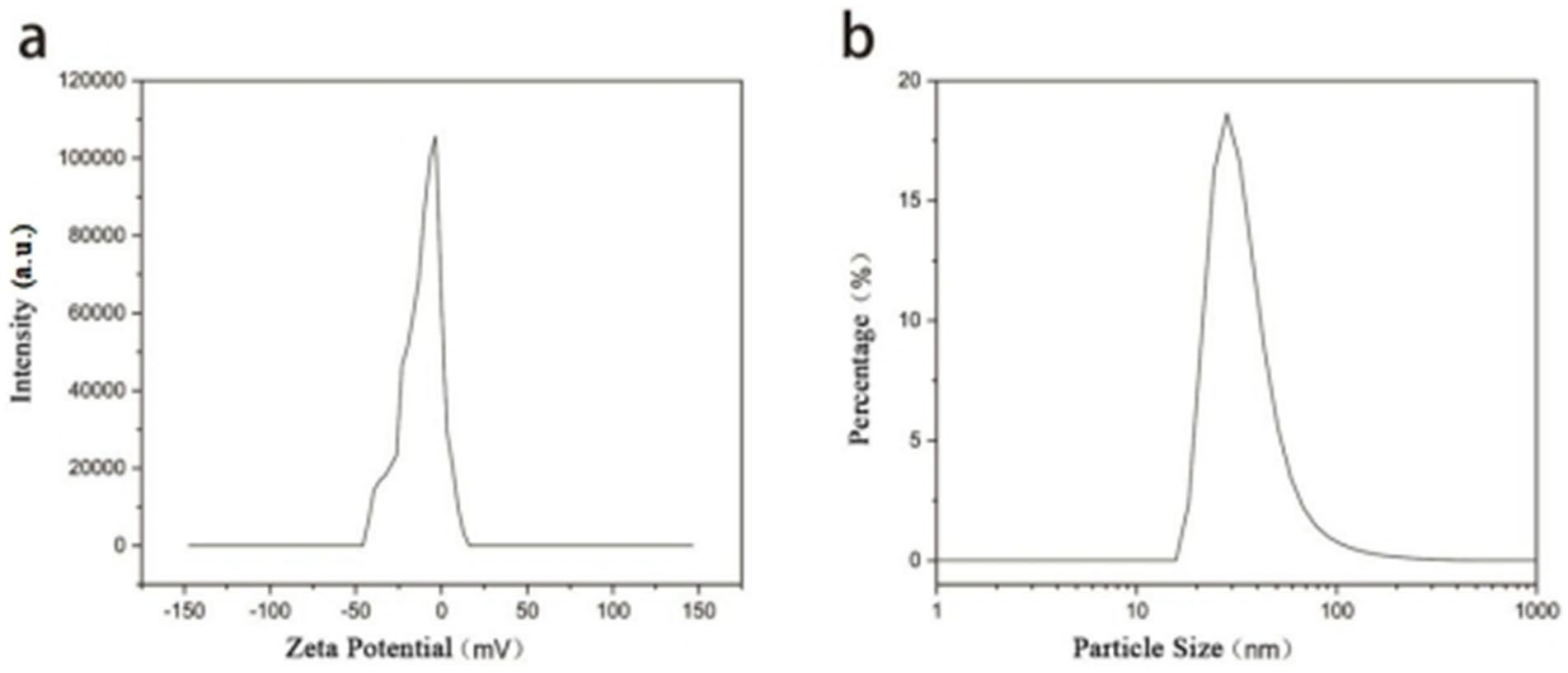
**(a)** Zeta potential distribution and **(b)** hydrodynamic particle size distribution of the synthesized AuNPs as measured by dynamic light scattering (DLS).

The Zeta potential of the synthesized gold nanoparticles (AuNPs) was measured to be −11.6 mV, with a sharp and narrow unimodal distribution, indicating relatively uniform surface properties among the particles. Dynamic light scattering (DLS) analysis revealed a hydrodynamic diameter distribution with the main peak located around 20–30 nm on a logarithmic scale, suggesting a concentrated size distribution and good monodispersity. The sharp peak shape without significant tailing further indicates minimal particle aggregation and a narrow size distribution, reflecting the favorable dispersibility of the synthesized AuNPs (Figure 4).

#### 3.3.3 Fourier-transform infrared spectroscopy (FTIR)

**Figure 5:**
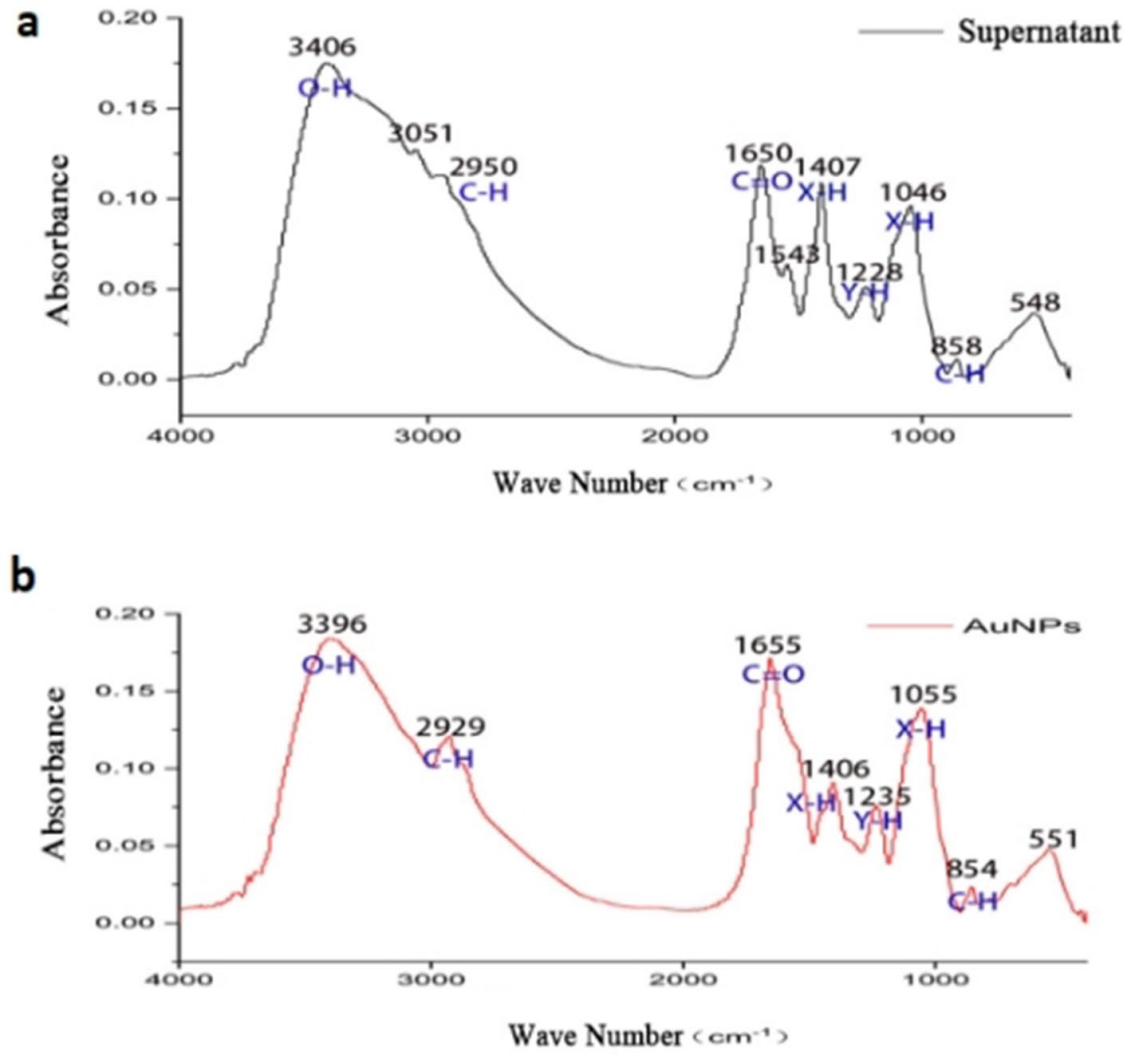
FTIR spectra of supernatant **(a)** and AuNPs **(b)**.

FTIR spectra (Figure 5) compared the functional groups in sterile supernatant and synthesized AuNPs. The supernatant exhibited absorption peaks at 3,406.19, 3,051.80, 2,950.77, 1,650.82, 1,543.79, 1,407.21, 1,228.23, 1,046.73, 858.54, and 548.56 cm^−1^. Peaks corresponding to O-H stretching (3,406.19 cm^−1^), C-H asymmetric stretching (3,051.80 and 2,950.77 cm^−1^), and C=O group absorption (1,650.82 cm^−1^) were observed. The FTIR spectrum of AuNPs lacked peaks at 3,051.80 cm^−1^ and 1,543.79 cm^−1^, indicating that these groups were not involved in reduction and stabilization processes.

## 4 Dose-dependent cytotoxicity of AuNPs across tumor and Normal cell lines

**Figure 6:**
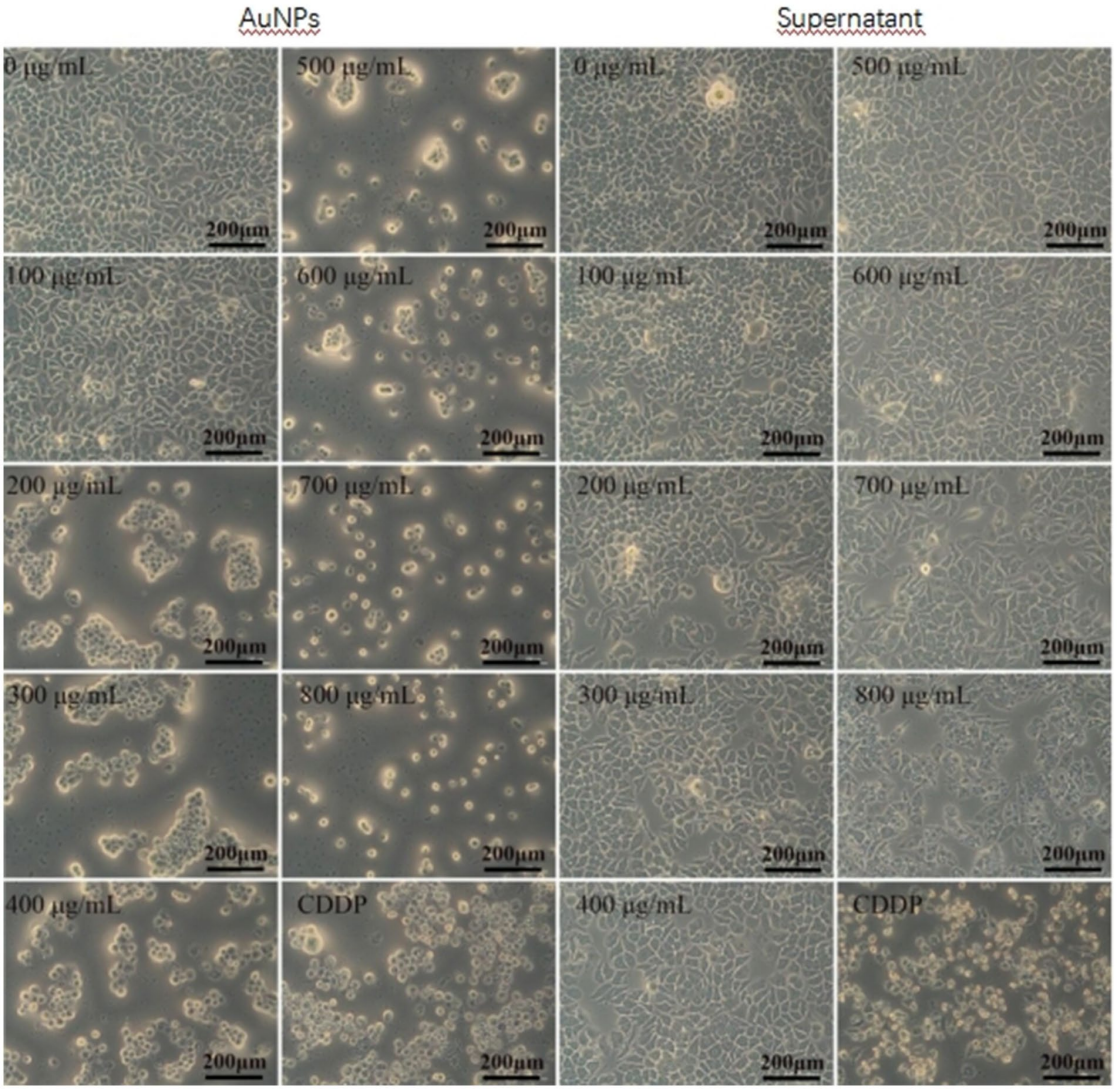
Microscopic images (40× magnification) showing the cytotoxic effects of gold nanoparticles (AuNPs), mycelial supernatant, and cisplatin (CDDP) on cervical cancer cells (HeLa).HeLa cells were treated with increasing concentrations (0–800 μg/mL) of AuNPs and supernatant, as well as with cisplatin as a positive control. Cell morphology changes, including cell shrinkage, rounding, and detachment, were observed with increasing concentration, especially at ≥500 μg/mL for AuNPs and ≥700 μg/mL for supernatant. CDDP treatment caused marked cytotoxic effects. All images were captured under 40× magnification; scale bar = 200 μm.

**Figure 7:**
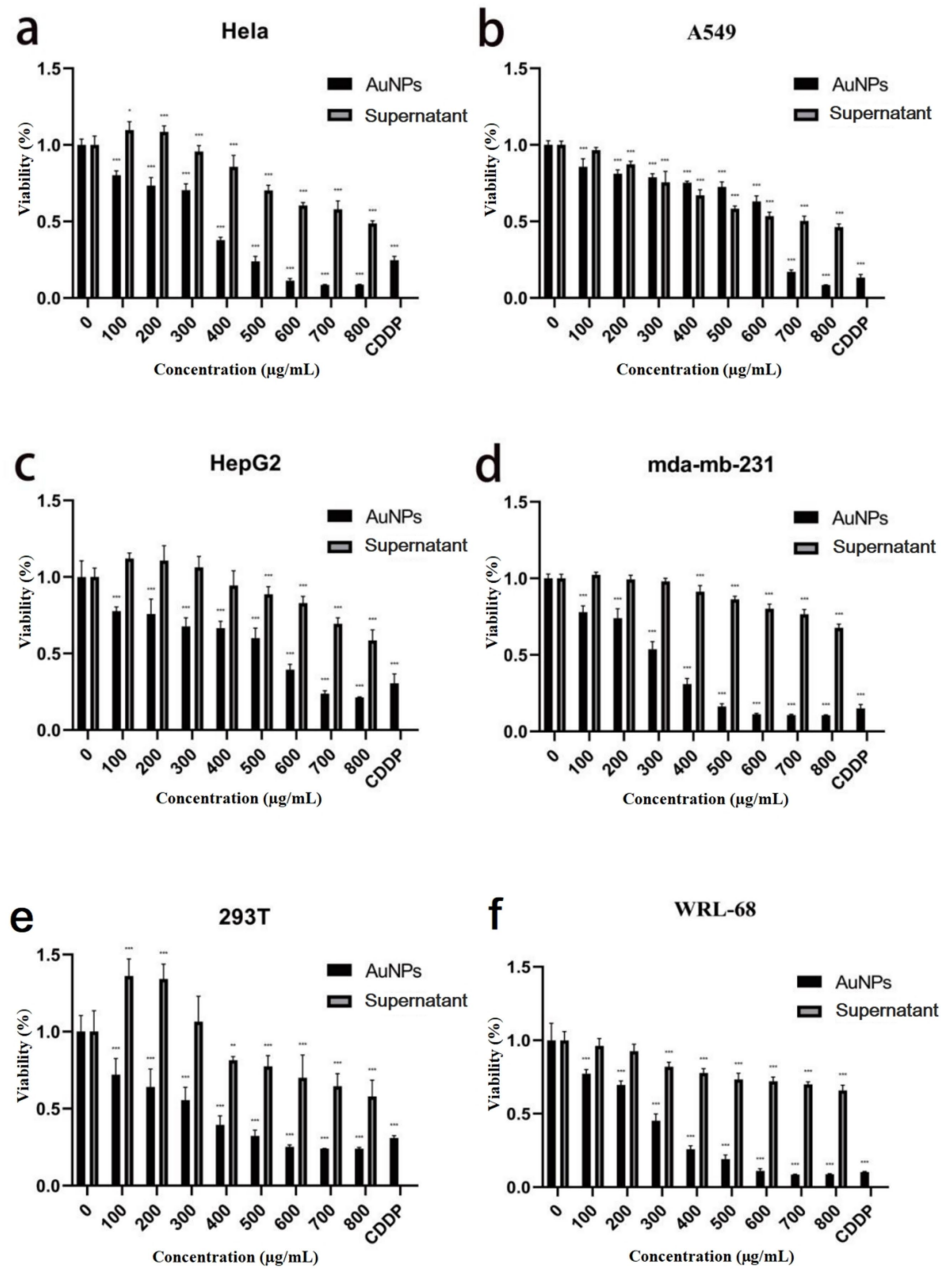
Bar graphs showing the cell viability of **(a)** cervical cancer cells (HeLa), **(b)** lung cancer cells (A549), **(c)** liver cancer cells (HepG2), **(d)** breast cancer cells (MDA-MB-231), **(e)** kidney cells (293T), and **(f)** liver cells (WRL-68) under treatment with AuNPs, sterile mycelial supernatant, and CDDP. *p < 0.05, **p < 0.01, ***p < 0.001.

With escalating concentrations of gold nanoparticles (AuNPs), significant morphological alterations were observed in MDA-MB-231 cells, characterized by roughened cell surfaces, vacuole formation, and decreased cell density (Figure 6). Comparable morphological changes were evident in the other three cell lines. MTT assay results (Figure 7) demonstrated variable toxicity of AuNPs across different cell types, with the concentration inducing maximum inhibitory activity differing among them. For the tumor cell lines MDA-MB-231, A549, and HepG2, peak toxicity occurred at 800 μg/mL, reducing cell viabilities to 10.60, 8.43, and 21.31%, respectively. The corresponding IC_50_ values were 314.89 ± 37.73 μg/mL, 638.84 ± 15.33 μg/mL, and 574.14 ± 35.94 μg/mL (Table 1). In contrast, maximum inhibition in HeLa cells was achieved at 700 μg/mL, with a viability of 8.57% and an IC_50_ of 378.95 ± 42.22 μg/mL (Table 1). Overall, AuNP exposure elicited a dose-dependent reduction in cell viability across all four cell lines. Figure 7 further reveals that the maximum inhibitory concentrations for the normal cell lines 293 T and WRL-68 were both 700 μg/mL, resulting in viabilities of 24.03 and 8.53%, respectively, with IC_50_ values of 386.64 ± 55.59 μg/mL and 244.905 ± 33.32 μg/mL (Table 1). These findings indicate that AuNPs exhibit dose-dependent toxicity toward both cell types, with a more pronounced effect observed in hepatocytes.

**Table 1:** IC_50_ values of AuNPs on tumor cells and normal cells.

	Hela	A549	HepG2	mda-mb-231	293 T	WRL-68
IC_50_ (ug/mL)	378.95 ± 42.22	638.84 ± 15.33	574.14 ± 35.94	314.89 ± 37.73	386.64 ± 55.59	244.91 ± 33.32

## 5 Impact of AuNPs on wound healing

**Figure 8:**
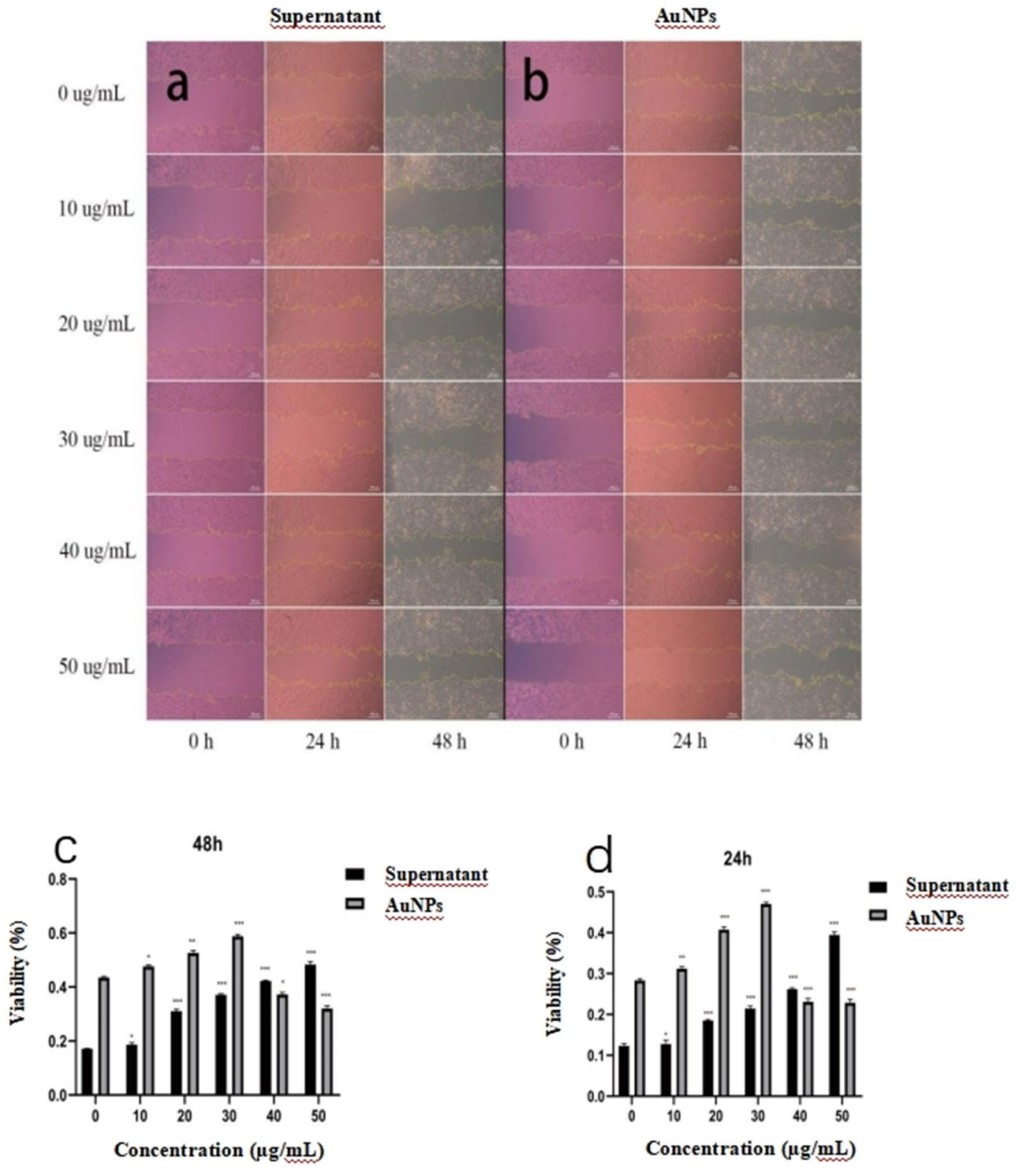
Microscopic images illustrating changes in wound area in WRL-68 cells following treatment with **(a)** sterile mycelial supernatant and **(b)** gold nanoparticles (AuNPs) **(c)** and **(d)** showing the wound healing rates of WRL-68 cells treated with gold nanoparticles (AuNPs) and sterile mycelial supernatant at 24 and 48 hours. *p < 0.05, **p < 0.01, ***p < 0.001.

Figure 8 illustrates the temporal changes in wound area during the healing process in response to sterile mycelial supernatant and gold nanoparticles (AuNPs). The data indicate a progressive reduction in wound area over time. As shown in Figures 8c,d, the wound healing rate for sterile mycelial supernatant exhibits a positive correlation with its concentration. For AuNPs, at concentrations ≤30 μg/mL, the wound healing rate increases with increasing concentration, peaking at 48 h with a healing rate of 58.07%, compared to 43.07% for the control group, demonstrating a significant enhancement of wound healing. Conversely, at concentrations >30 μg/mL, the healing rate falls below that of the control group, indicating an inhibitory effect. This inhibition is most pronounced at 50 μg/mL, where the healing rate at 48 h is reduced to 31.01%, compared to 43.07% for the control. Furthermore, at concentrations ≤30 μg/mL, AuNPs consistently demonstrate superior wound healing rates and bioactivity compared to sterile mycelial supernatant.

## 6 Antioxidant activity of AuNPs

**Figure 9:**
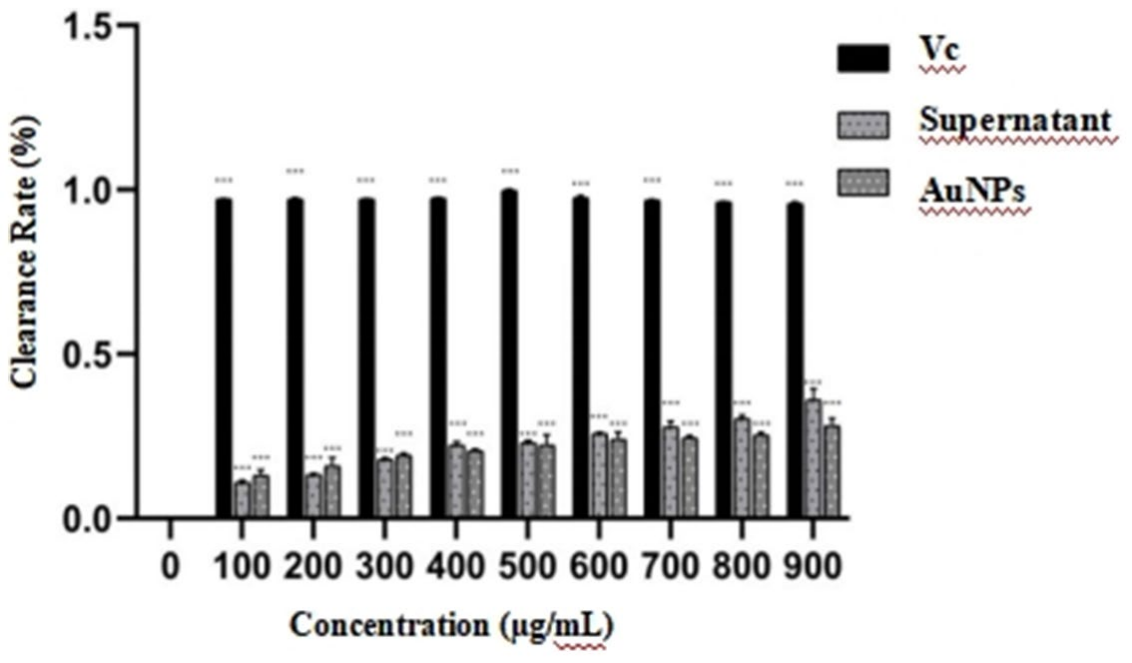
Representative images illustrating the DPPH free radical scavenging effects of gold nanoparticles (AuNPs), sterile mycelial supernatant and vitamin C. *p < 0.05, **p < 0.01, ***p < 0.001.

In this study, observations from Figure 9 indicate that, relative to the blank group, the liquid in the wells of the AuNP group exhibited a deepening color with increasing concentration, attributable to the intrinsic coloration of the AuNP solution. However, no visually discernible differences in DPPH scavenging activity were observed in this group. Likewise, the sterile mycelial supernatant group showed no notable color changes detectable by the naked eye. In contrast, the vitamin C treatment group displayed a distinct color transition from purple to pale yellow, signifying substantial DPPH scavenging activity. Quantitative analysis, presented as a bar chart in Figure 9, reveals that within the 0–900 μg/mL concentration range, the antioxidant activity of both sterile mycelial supernatant and AuNPs increased with concentration, reaching maxima of 23.74 and 32.14%, respectively, at 900 μg/mL. The corresponding IC_50_ values were 2,908.32 μg/mL and 12,419.00 μg/mL. Nonetheless, the antioxidant capacities of these compounds were markedly inferior to that of vitamin C.

## 7 Discussion

The optimization experiments for gold nanoparticle (AuNP) synthesis revealed that synthesis efficiency did not increase with escalating gold ion concentrations as expected. Notably, ultraviolet (UV) absorption peaks were detected at 0.5 and 1.0 mM, with the latter exhibiting greater absorption intensity than the former. This indicates that within the lower concentration range, elevated gold ion levels enhance AuNP synthesis, resulting in increased UV–Vis absorbance. Conversely, at higher concentrations, particle aggregation or size alterations occur, causing a shift in the UV–Vis spectrum (Valsalam et al., 2019; Sathiyapriya et al., 2024). A similar trend was observed with mycelial resuspension time, where UV intensity at 72 h was lower than at 48 h, suggesting a decline in the activity of bioactive compounds involved in synthesis, thereby reducing efficiency. The pH of the reaction system, a well-established determinant of nanoparticle synthesis and stability, was found to be optimal at 6.25 under weakly acidic conditions, promoting AuNP formation. Stronger acidity impaired synthesis efficiency, likely due to diminished activity of bioreducing biomolecules in highly acidic environments (Ahmed et al., 2020; Patel et al., 2025). Although strong alkaline conditions supported AuNP synthesis, they induced a redshift in the UV absorption peak, indicative of increased particle size or aggregation (Hu et al., 2019; Krishnan and Lakshmanan, 2024). Supernatant concentration also influenced AuNP synthesis, with undiluted sterile mycelial supernatant yielding the highest UV absorption peak intensity. However, increasing supernatant concentration led to a redshift in the UV absorption peak of synthesized AuNPs, suggesting the formation of larger particles. This implies that supernatant concentration affects both AuNP yield and properties, consistent with Britto et al. (2017).

Morphological attributes of nanoparticles, including texture, size, and shape, serve as critical descriptors, with size being a key factor in biological activity. Chen et al. (2022) demonstrated that smaller nanoparticles enhance antitumor efficacy by improving cellular penetration. AuNPs show substantial potential in cancer therapy, applicable to lung, breast, uterine, and colorectal cancers. This study assessed the *in vitro* cytotoxicity of AuNPs on tumor and normal cell lines, observing morphological changes such as cell shrinkage and budding post-treatment. A dose-dependent decline in cell viability was evident across both cell types, lacking the selective toxicity toward tumor cells reported by Lee et al. (2015). Tumor cells exhibited greater tolerance to AuNP toxicity than normal cells, a difference attributed to interactions between nanoparticles and cell surface biomolecules (Adabi et al., 2017). The intensity of these interactions is governed by nanoparticle physicochemical properties (e.g., shape, size, surface chemistry), cellular features (e.g., membrane interactions, protein adsorption, permeability, stability in biofluids) (Maffre et al., 2011), and factors such as concentration and exposure duration (Muthukumar et al., 2016; Pan et al., 2007; Das and Velusamy, 2014), contributing to variable toxicity profiles.

we performed FTIR analysis to compare the functional groups present in the sterile mycelial supernatant and the synthesized AuNPs. The FTIR spectra revealed peaks corresponding to O–H stretching (broad peak around 3,400 cm^−1^), N–H bending (approximately 1,640 cm^−1^), and C=O stretching vibrations, suggesting the presence of hydroxyl, amine, and carbonyl groups. These functional groups are commonly found in biomolecules such as proteins, peptides, and other secreted metabolites. Although we did not carry out proteomic or metabolomic profiling such as GC–MS or LC–MS in the present work, similar studies have attributed the reduction and capping of gold ions to proteins, enzymes, and secondary metabolites secreted by Streptomyces and related actinomycetes (e.g., Ahmed et al., 2020; Narayanan and Sakthivel, 2011).

In wound healing experiments, microscopic analysis and data indicated that AuNP concentrations <30 μg/mL significantly enhanced healing at 24 and 48 h. Compared to the untreated control, treated groups showed a notable reduction in wound area, with a concentration-dependent decrease up to 30 μg/mL, corroborating findings by Hu et al. (2019). However, at concentrations of 30–50 μg/mL, wound healing rates declined relative to the control, with the reduction magnitude increasing with concentration, suggesting inhibition of cell proliferation and healing. Zgheib et al. (2019) suggested that nanoparticles may promote healing via increased collagen formation and fibrotic factor regulation, while Boomi et al. (2020b) proposed mechanisms involving alterations in cell membrane potential, ATPase inhibition, or moderate reactive oxygen species (ROS) induction.

Regarding antioxidant activity, the DPPH radical scavenging assay showed that the biosynthesized gold nanoparticles (AuNPs) in this study exhibited moderate free radical scavenging capacity, which increased notably with concentration, consistent with the findings of Ahn et al. (2018). Specifically, the IC₅₀ value of the AuNPs synthesized here was 12,419.00 μg/mL, significantly higher than that of AuNPs synthesized using mango seed extract (IC₅₀ = 256 μg/mL) reported by Donga et al. (2020). This difference is likely due to variations in the types and amounts of bioactive molecules coating the nanoparticle surfaces, which play a crucial role in antioxidant capacity (Markus et al., 2017; Patra et al., 2016). For example, plant extracts are rich in phenolics, flavonoids, and other reducing functional groups that effectively scavenge free radicals by donating hydrogen atoms or electrons (Tang et al., 2015). The antioxidant mechanism of AuNPs primarily derives from the bio-molecules coating their surfaces; these natural reducing agents not only stabilize the nanoparticles but also directly participate in electron transfer reactions, neutralizing oxidative free radicals *in vivo* and *in vitro*, thereby reducing oxidative stress-induced cellular damage (Sharma et al., 2019). Moreover, nanoparticle size and morphology influence antioxidant efficiency, with smaller nanoparticles having larger specific surface areas, facilitating more effective interactions with free radicals (Huang et al., 2020). It is noteworthy that AuNPs synthesized by different methods exhibit significant differences in surface chemistry, further affecting their efficacy in free radical scavenging. Although the AuNPs synthesized in this study demonstrated antioxidant activity, their relatively high IC₅₀ suggests room for improvement in free radical scavenging ability. Recently, surface functionalization has been widely applied in nanomaterial research, introducing molecules with antioxidant properties (such as polyphenols and vitamin C) to enhance the nanoparticles’ antioxidant performance, biocompatibility, and targeting ability (Kumar et al., 2021; Li et al., 2022). Therefore, future research should focus on chemically or biologically modifying the AuNP surface to improve their antioxidant capacity, thereby expanding their biomedical applications, particularly in anti-inflammatory, anticancer, and chronic disease treatments (Singh et al., 2020). In summary, the antioxidant activity of AuNPs in this study is moderate but provides a foundation for their potential use as biomedical materials, with surface functionalization strategies promising to further enhance their clinical application value.

## 8 Conclusion

This study successfully achieved the biosynthesis of AuNPs using extracellular secretions from YJD8, with optimized reaction conditions and comprehensive evaluation of their cytotoxicity. Future research should explore surface functionalization or conjugation with specific targeting ligands to enhance cellular selectivity and minimize off-target toxicity, thereby facilitating broader and more effective applications in cancer therapeutics.

## Data Availability

The original contributions presented in the study are included in the article/supplementary material, further inquiries can be directed to the corresponding authors.
